# Integrated bioinformatic analyses investigate macrophage-M1-related biomarkers and tuberculosis therapeutic drugs

**DOI:** 10.3389/fgene.2023.1041892

**Published:** 2023-02-08

**Authors:** Siqi Deng, Shijie Shen, Keyu Liu, Saeed El-Ashram, Abdulaziz Alouffi, Beniamino Terzo Cenci-Goga, Guomin Ye, Chengzhang Cao, Tingting Luo, Hui Zhang, Weimin Li, Siyuan Li, Wanjiang Zhang, Jiangdong Wu, Chuangfu Chen

**Affiliations:** ^1^ Key Laboratory of Xinjiang Endemic and Ethnic Diseases Cooperated by Education Ministry with Xinjiang Province, Shihezi University, Shihezi, China; ^2^ Faculty of Science, Kafrelsheikh University, Kafr El-Sheikh, Egypt; ^3^ King Abdulaziz City for Science and Technology, Riyadh, Saudi Arabia; ^4^ Department of Veterinary Medicine, University of Perugia, Perugia, Italy; ^5^ Beijing Chest Hospital, Capital Medical University, Beijing, China

**Keywords:** tuberculosis, WGCNA, CIBERSORT, polarization, macrophage-M1, biomarkers, drug prediction, enrichment analysis

## Abstract

Tuberculosis (TB) is a common infectious disease linked to host genetics and the innate immune response. It is vital to investigate new molecular mechanisms and efficient biomarkers for Tuberculosis because the pathophysiology of the disease is still unclear, and there aren’t any precise diagnostic tools. This study downloaded three blood datasets from the GEO database, two of which (GSE19435 and 83456) were used to build a weighted gene co-expression network for searching hub genes associated with macrophage M1 by the CIBERSORT and WGCNA algorithms. Furthermore, 994 differentially expressed genes (DEGs) were extracted from healthy and TB samples, four of which were associated with macrophage M1, naming RTP4, CXCL10, CD38, and IFI44. They were confirmed as upregulation in TB samples by external dataset validation (GSE34608) and quantitative real-time PCR analysis (qRT-PCR). CMap was used to predict potential therapeutic compounds for tuberculosis using 300 differentially expressed genes (150 downregulated and 150 upregulated genes), and six small molecules (RWJ-21757, phenamil, benzanthrone, TG-101348, metyrapone, and WT-161) with a higher confidence value were extracted. We used in-depth bioinformatics analysis to investigate significant macrophage M1-related genes and promising anti-Tuberculosis therapeutic compounds. However, more clinical trials were necessary to determine their effect on Tuberculosis.

## Introduction

Tuberculosis (TB) is still a chronic, airborne infectious disease that is the leading cause of death in adults worldwide (Churchyard et al., 2017; Natarajan et al., 2020). It is characterized by continuing inflammation of the lung tissue caused by *Mycobacterium tuberculosis* (Mtb) (Muefong and Sutherland 2020). Recent WHO surveys found that one-third of the world’s population was latently affected by tuberculosis, and over 1.5 million patients die annually. Clinical symptoms, radiography, the tuberculin skin test (TST), and the interferon-gamma release assay (IGRA) are the most critical components of a patient’s diagnosis (Shimizu and Mori 2017). Pharmacotherapy is recommended as the initial treatment for tuberculosis patients (Suarez et al., 2019). Simultaneously, public health approaches have effectively mitigated the rapid rise in TB patients and saved millions of lives worldwide (Guinn and Rubin 2017). Mtb persistent spread, however, contributes to the emergence and evolution of drug-resistant strains, which include multidrug-resistant tuberculosis (MDR-TB) and extensively drug-resistant tuberculosis (XDR-TB) (Migliori et al., 2020). It is implied that distinct steps must be taken to discover the potential molecular mechanism of the host’s immune defense against Mtb. In the meantime, effective diagnostic biomarkers and promising therapeutic compounds should be retrieved to aid in the early diagnosis and treatment of tuberculosis (TB). According to statistics, only 10% of latent Mtb infections develop into active diseases (Bloom et al., 2017). The individual immune response plays a crucial role in determining the progression and outcome of infection (Chai et al., 2020).

Shim et al. (2020) discovered that Mtb induced the early stage of the inflammatory response, resulting in granuloma formation, which increased the recruitment of immune cells, such as macrophages, neutrophils, natural killer (NK) cells, dendritic cells (DCs), B cells, T cells, etc (Korb et al., 2016). Additionally, macrophages are recognized as Mtb host cells, and their polarization is crucial to their role in immune surveillance (Zhou et al., 2019). Macrophages were often divided into the M0, M1, and M2 subtypes. The polarized macrophages are often preceded by the resting-state macrophage (M0). Macrophage M1 is activated during bacterial infection, and M2 is associated with the anti-inflammatory response (Wang et al., 2021; Zhang et al., 2022). According to a previous study, macrophage M1 is more effective than M2 in suppressing intracellular Mtb and protecting cells (Mills 2012). Mycobacteria escape, however, is often accomplished *via* dysregulating macrophage polarization (Le Y et al., 2020; Mily et al., 2020). Overall, it is crucial to identify macrophage M1-related biomarkers that might facilitate the discovery of their influence on the immunological pathogenesis of Mtb.

Bioinformatics tools have improved dramatically over the last several decades, allowing researchers to quickly and easily validate new biomarkers and develop major signal pathways after Mtb infection. A comprehensive bioinformatics study has validated associations between TB and genes, including IFIT1, CCR7, and GPR84 (Li et al., 2020; Deng et al., 2021). To mine the association between hub gene modules and clinical characteristics, we adopt weighted gene co-expression network analysis (WGCNA) to construct co-expression modules (Nangraj et al., 2020). It is often used to discover new biomarkers at the transcriptional level (Zhang et al., 2021).

In our study, we combined two datasets with 46 TB and 73 healthy blood samples to look for a novel biomarker in TB *via* WGCNA. Estimating Relative Subsets of RNA Transcripts (CIBERSORT) was also used to determine immune cell type and calculate the level of infiltration of different immune cells (Chen et al., 2018). Thus, the best significant macrophage M1-related modules were selected, and key genes in hub modules were validated and tested externally. Additionally, we used the Connectivity Map (CMap) online database to predict target chemicals that have a beneficial impact on TB. The above analysis overcame conventional analysis’s limitations and offered a fresh perspective on the molecular diagnosis of TB.

## Materials and methods

### Gene expression data

GSE19435, GSE83456, and GSE34608 tuberculosis blood mRNA expression profiles were downloaded from NCBI-GEO (https://www.ncbi.nlm.nih.gov/geo/) in turn. The GPL6947 platform was used to extract the data from the GSE19435 microarray profiles, which included 21 TB and 12 health samples (Berry et al., 2010). The GSE83456 microarray profiles were generated using the GPL10558 platform and included 45 TB and 61 matched control samples (Blankley et al., 2016). GSE34608 was produced using the GPL6480 platform and included 18 healthy and 8 TB samples. GSE34608 was normalized using the R software package “limma” (Maertzdorf et al., 2012). In addition, we combined GSE19435 and GSE83456 microarray profiles, and the “sva” and “limma” packages were used to batch an integrated microarray profile normalization (Liu et al., 2021). GSE34608 was used as an external dataset, while GSE19435 and GSE83456 were used as training datasets.

### Identification the level of immune cell infiltration and construction co-expression networks

Using mRNA expression data, the CIBERSORT algorithm calculated the proportion of each sample’s 22 types of immune cells (Sui et al., 2020). In the current study, GSE19435 and GSE83456 were subjected to the CIBERSORT algorithm using the R package to pool distinct immune cells of tissue into corresponding subsets. Then, in 66 TB samples with coefficients of variation greater than 0.07, we chose the significant variant gene. The R package “WGCNA” produced a weighted gene co-expression network from 2,413 genes (Wang et al., 2020). Furthermore, Pearson’s correlation matrices were identified by converting the expression of study object transcripts into a similarity matrix. And then, transferring an adjacency matrix from the similarity matrix, as quantified by amn = |cmn|β (anm = adjacency between paired genes; cmn = correlation Pearson’s coefficient between paired genes; β = soft-power threshold) (Cui et al., 2021). We can suppress weak correlation and increase strong correlation of genes by changing the parameter β (Lin et al., 2020). The adjacency matrix was converted into a topological overlap matrix after a cutoff point (power of β= 4) was determined. We used dynamic hybrid cutting to group genes with similar expressions into distinct modules. The bottom-up algorithm was run with a cutoff point of 30 for module minimum size. The identical modules were then merged using shear height = 0.25.

### Determination macrophage M1-related hub module and genes

Individual modules were subjected to constituent analysis using module eigengenes (Wang et al., 2008). To identify the hub modules, the Pearson test was used to assess macrophage infiltration and the relevance of gene modules. *p* < 0.05 was used to identify a significant module associated with macrophage-M1. The macrophage M1-related subtype and module with the highest correlation coefficient were designated as the hub module for further study. After hub gene removal, it is critical to evaluate module connectivity and phenotype (clinical traits) (Zhang et al., 2022). The absolute value of the correlation between a gene’s expression profile and clinical traits is referred to as gene significance (GS). Module membership is defined as the correlation between a module’s eigengene and the expression profile of a gene (Wan et al., 2018). The hub genes in the hub module were extracted using an exact cutoff value (gene significance value > 0.5 and model membership value > 0.8) (Cui et al., 2021).

### GO and KEGG pathway analysis and PPI network construction of hub genes

We performed KEGG and Gene Ontology (GO) enrichment analyses after compiling the list of hub genes, and the PPI network was then generated. R software “org.Hs.eg.db” was utilized to convert gene symbols into gene IDs, and then “clusterProfiler” was employed to determine significant items with a cutoff criterion (*p* < 0.05 and false discovery rate–adjusted *p* values (FDR) < 0.05) (Deng et al., 2021). We sorted the GO and KEGG pathways by the maximum number of genes and then screened the top ten corresponding items. The identified GO, and KEGG items were uploaded to “ggplot2” for visualization and merging hub gene enrichment analysis. STRING’s online website (https://cn.string-db.org/) was used to determine the encoded protein interaction of hub genes using the cutoff value (interaction score >0.4) (Han et al., 2020). These protein interaction data were submitted to Cytoscape to build and modify the PPI network.

### Prediction of key diagnostic markers for tuberculosis

To screen DEGs in training datasets, the expression data of normalized datasets were submitted to R software “limma” with a cutoff point (FDR< 0.05 and |log fold change (FC)|≥0.5) (Huang et al., 2021). Identified DEGs were used to reveal the underlying molecular mechanism by GO and KEGG analysis following the instructions mentioned earlier, and the top 10 GO and KEGG terms were to be visualized directly by R package “ggplot2” and “GOplot.” And then, “ggplot2” and “pheatmap” were orderly employed to visualize the DEGs in the TB group. Moreover, DEGs were imported into the STRING online tool to obtain the protein interaction information. The Cytoscape software MCODE plug-in was utilized to identify the top module with an exact cutoff condition (degree cutoff = 2, node score cutoff = 0.2, k-core = 2, and max depth = 100) (Deng et al., 2021). The overlapping genes of DEGs and hub genes were defined as candidate key genes, determined by the R package “VennDiagram.” In addition, GSE34608 is an external dataset for further expression profile analysis. ROC analysis was executed by “pROC” to estimate the diagnostic value of key genes between TB and the control group.

### Total RNA extraction and quantitative real-time PCR analysis

The TRIzol reagent (Invitrogen, United States) was used to extract total RNA according to the manufacturer’s instructions. Then, using a PrimeScript RT Reagent Kit, RNA was reverse-transcribed into cDNA (TransGen Biotech, China). The cDNA amplification was conducted by QuantStudio Real-Time PCR Systems (Thermo Fisher Scientific, United States). The primer sequences of RTP4, CXCL10, CD38, and IFI44 were:

RTP4-F:5′- ACA​TGG​ACG​CTG​AAG​TTG​GAT-3′,

RTP4-R: 5′-TAC​GTG​TGG​CAC​AGA​ATC​TGC-3′;

CXCL10-F: 5′- AGT​GGC​ATT​CAA​GGA​GTA​CC -3′,

CXCL10-R: 5′- GCA​ATG​ATC​TCA​ACA​CGT​G -3′;

CD38-F: 5′-CAA​CTC​TGT​CTT​GGC​GTC​AGT-3′,

CD38-R: 5′-CCC​ATA​CAC​TTT​GGC​AGT​CTA​CA-3′;

IFI44-F: 5′-ATG​GCA​GTG​ACA​ACT​CGT​TTG-3′;

IFI44-R: 5′-GCA​ACT​GGA​CCC​TGT​CGT​T-3′.

### Small molecular therapeutic chemicals detection in CMap

It has been shown that the Broad Institutes Connectivity Map (CMap) (https://clue.io/) is a valuable open database for identifying potential new tuberculosis small-molecule therapeutic agents and examining the underlying mechanisms of physiological processes and action (Kapoor et al., 2016; Vanderstocken et al., 2018). Significant DEGs were submitted to the CMap database in the current study to conduct enrichment analysis using the cutoff criteria (0.80<|connectivity score|<1 and *p* < 0.05). The compounds with substantial enrichment value likely had an underlying therapeutic impact on TB. Finally, PubChem (https://pubchem.ncbi.nml.gov) was used to export the 3D structures of small molecule compounds (Kim et al., 2016).

### *M. tuberculosis* culture

*M. tuberculosis*-BCG was obtained from Shihezi University (Shihezi, China). BCG was grown in Middlebrook 7H9 medium (Sigma-Aldrich, United States) containing 5% glycerol and 10% oleic albumin dextrose (ADC). Rapidly growing BCG was harvested and suspended in a bacterial culture medium after centrifugation at 3,200 rpm for 10 min.

### Cell culture and *M. tuberculosis* infection

The monocytic THP-1 cell line was purchased from Procell (Wuhan, China). Before use, cells were seeded in a 6-well plate with a concentration of 2 × 10^6^ cells/well in RPMI 1640 medium (Gibco, United States) supplemented with 10% FBS (Gibco, United States) and 100 ng/mL phorbol 12-myristate 13-acetate (PMA) for 48 h at 37°C with 5% CO2 to induce adherent and differentiated macrophages. Images were acquired using a NIS system under a light microscope (Nikon, ECLIPSE Ti, Japan). Differentiated THP-1 was considered the best condition when infected for 4 h with a multiplicity of infection (MOI) of 10. The extracellular bacteria were washed with phosphate buffer saline (PBS, Solarbio, China). THP-1 infected cells were cultured for 24 h.

### Statistical analysis

R 4.2.1 software was used for statistical analysis. All experiment results were presented as mean ± SD and analyzed using an unpaired two-tailed Student’s *t*-test. And *p*-value < 0.05 was used to denote statistical significance. The Mann-Whitney U test was used to compare the expression of key genes between the healthy and TB groups.

## Result

### mRNA expression data

**FIGURE 1:**
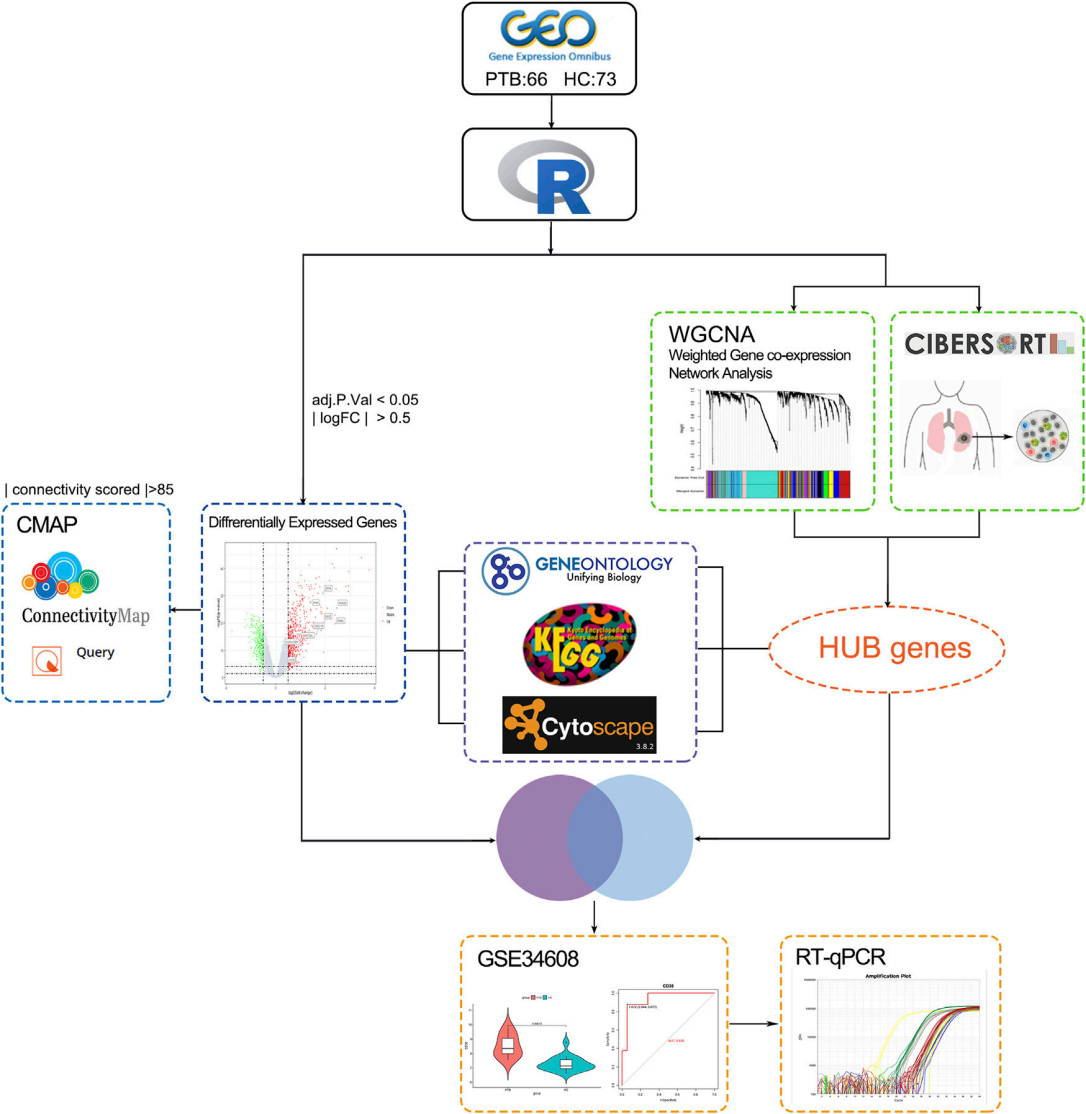
The study workflow. TB: tuberculosis; GEO: Gene expression omnibus; GO: genes ontology; KEGG: Kyoto Encyclopedia of Genes and Genomes; PPI: protein-protein interaction network; CMap: connectivity Map; CIBERSORT: Estimating Relative Subsets ff RNA Transcripts; WGCNA: weighted gene co-expression network analysis; DEGs: Differentially expressed genes; qRT-PCR; quantitative real-time PCR analysis; ROC curve: receiver operating characteristic curve.

Figure 1 depicts the study design utilized in this research study. Two mRNA expression profiles (GSE19435 and GSE83456) were extracted from the NCBI GEO database for bioinformatics analysis. GSE19435 and GSE83456 contain 66 TB samples and 73 normal samples. All sample data from gene profile datasets were combined and bath-normalized before bioinformatics analysis (Supplementary Figures S1A, B).

### Immune-infiltration level analysis and establishment of the co-expression network

CIBERSORT is a well-known online tool that uses gene expression arithmetic to estimate the constituents of immune cells in pathological tissue (Guan et al., 2022). The CIBERSORT algorithm differs from traditional deconvolution, because it is based on a precise analysis of unspecified data and noise in infiltrating immunity (Kawada et al., 2021). The immune cell composition of tuberculosis patients is unknown. For each sample, we used the analytical algorithm CIBERSORT to estimate the abundance of 22 cell subpopulations. Three macrophage subtypes in TB tissues were selected as trait data for WGCNA analysis (Supplementary Table S1).

**FIGURE 2:**
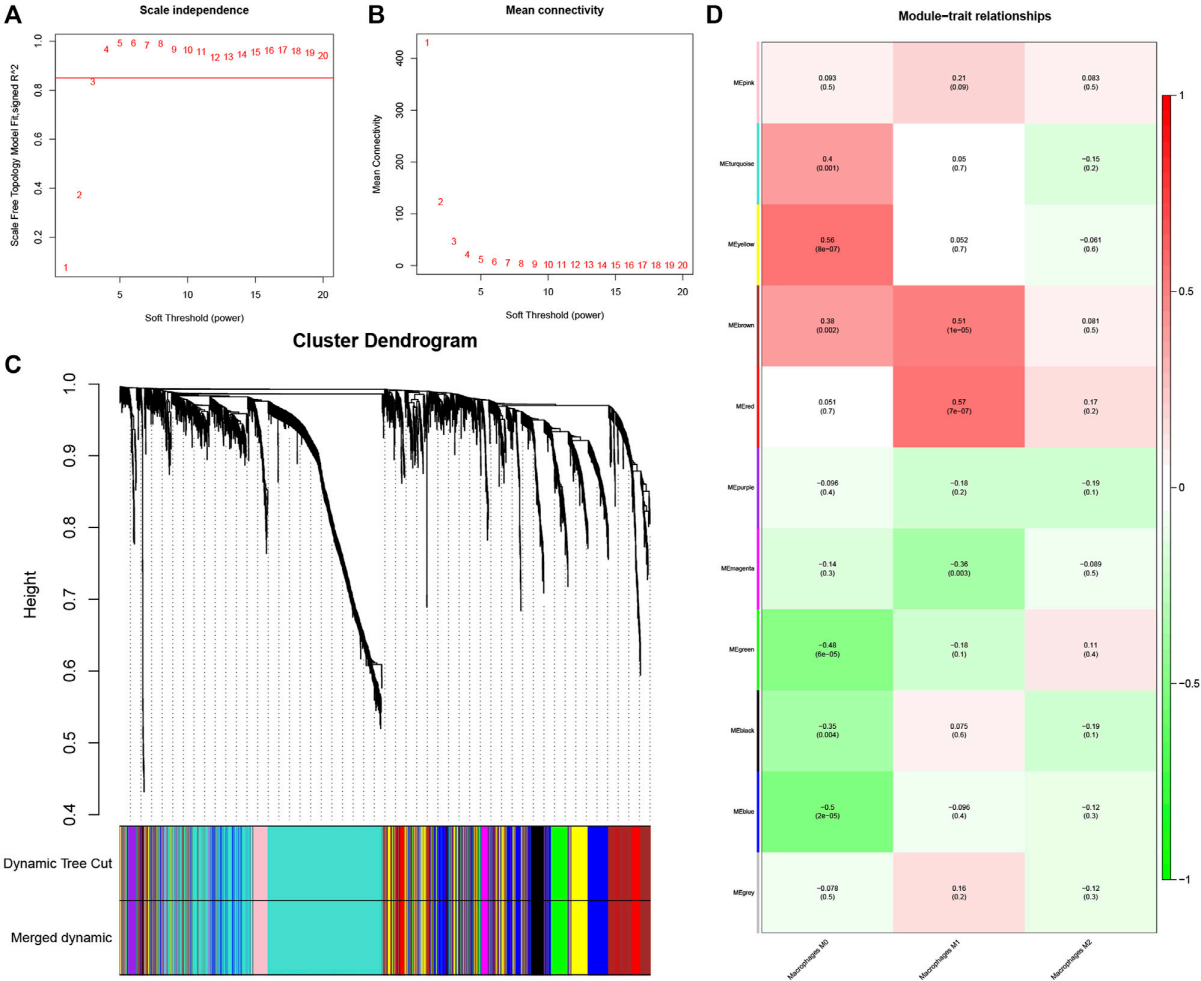
Determination of hub modules in TB. **(A)** Identification of the scale independence index of the 1–20 soft threshold power (β = 4). **(B)** Verification of the mean connectivity of 1–20 soft threshold power. **(C)** Hierarchical clustering divided genes into distinct modules that presented as different colors. **(D)** Heatmap presents relation of module eigengenes with macrophages M1 infiltration.

The gene co-expression network is increasingly used to study gene system-level function (Zhang and Horvath 2005). The weighted gene co-expression network (WGCNA) is commonly used to discover latent correlations between gene expression data and phenotypic traits. It is distinguished by increasing the computation network’s dimension and maintaining a topological network with a free scale (Toubiana et al., 2019). To establish the co-expression network, 2,413 genes with correlation coefficients greater than 0.07 were identified as variant genes (Supplementary Figure S1C; Supplementary Table S2). We developed a scale-independent topological network with *R* ^2^ = 0.85 (β = 4) soft thresholding power (Figures 2A, B.

The dynamic hybrid cutting method was then used to generate a hierarchical clustering tree. Prior research has shown that a single gene is a leaf on the tree, and the branches of the hierarchical clustering dendrogram correspond to a group of genes with similar biological significance (Dong and Horvath 2007; Hou et al., 2021). As for macrophage-related modules, 11 gene modules were identified (Figure 2C).

**FIGURE 3:**
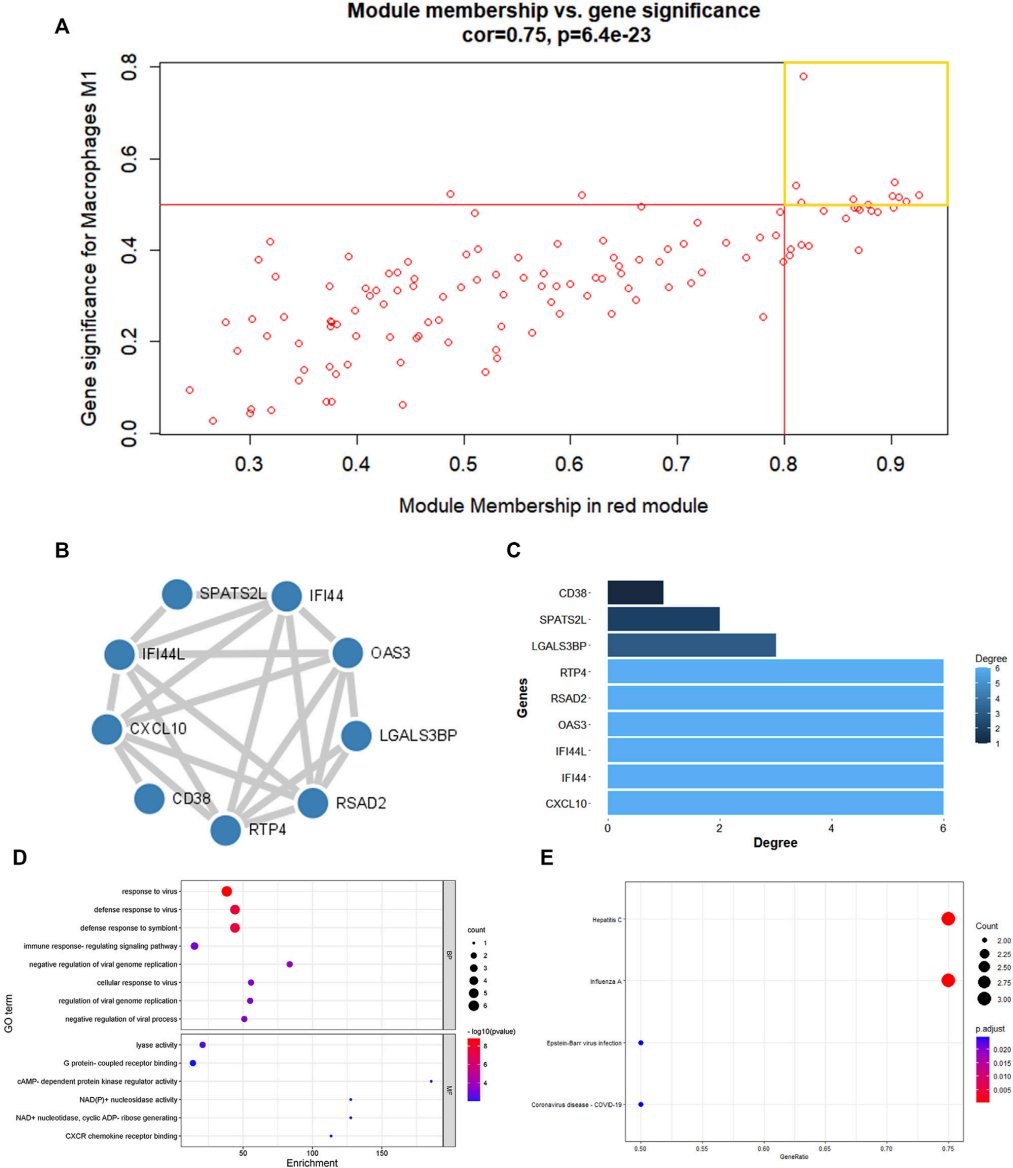
PPI network establishment and enrichment analysis of hub genes. **(A)** A scatter diagram of genes in red modules. Each red dot stood for a gene, spot inside the yellow box implied genes of Module Membership >0.8 and Gene Significance >0.5. **(B)** PPI network were construction and circular nodes stood for hub genes. **(C)**Bar graph shown the number of protein interactions in PPI network. **(D)** The GO items of hub genes. **(E)**The KEGG pathway of hub genes.

The red and brown modules closely related to Mϕ1 had *R* ^2^ = 0.57 and *R* ^2^ = 0.51, respectively. With Mϕ0 *R* ^2^ = 0.56, the yellow module demonstrated high relevance. Other modules with *R* ^2^ values less than 0.5 were found to be insignificant. The red module was chosen as the hub module with the best connectivity (R2 = 0.57, p = 7e−07) (Figure 2D). With precise cutoff criteria (module membership values >0.8 and gene significance values >0.5), 9 of 120 genes from the hub module were identified as hub genes (Figure 3A; Supplementary Table S3).

### GO and KEGG enrichment analysis of hub module

The main feature of GO analysis is mining the correlation between genes by annotating and categorizing gene sets based on biological process (BP), molecular function (MF), and cellular component (CC) (Dalmer and Clugston 2019). KEGG is an extensive database that integrates genomic, chemical, and system function data (Kanehisa et al., 2017). The enrichment analysis results revealed that GO and KEGG terms were chosen as significant terms using an exact criterion (*p* < 0.05 and FDR<0.05). Hub genes were primarily associated with “response to virus,” “defense response to virus,” “defense response to symbiont,” “immune response-regulating signaling pathway,” “cellular response to virus,” “regulation of viral genome replication,” “negative regulation of viral genome replication,” and “negative regulation of viral process,” according to the GO-BP terms. Significant GO-MF terms showed that hub genes were primarily involved in “lyase activity,” “cAMP-dependent protein kinase regulator activity,” “NAD(P)+ nucleosidase activity,” “NAD + nucleotidase, cyclic ADP-ribose generating,” “G protein-coupled receptor binding” and “CXCR chemokine receptor binding” (Figure 3D; Table 1).

**TABLE 1:** Gene ontology analysis of Hub genes.

Category	Term	Count	Gene symbol
BP	GO:0009615∼response to virus	6	RTP4/RSAD2/OAS3/IFI44L/IFI44/CXCL10
BP	GO:0051607∼defense response to virus	5	RTP4/RSAD2/OAS3/IFI44L/CXCL10
BP	GO:0140546∼defense response to symbiont	5	RTP4/RSAD2/OAS3/IFI44L/CXCL10
BP	GO:0002764∼immune response-regulating signaling pathway	3	RSAD2/OAS3/CD38
BP	GO:0045071∼negative regulation of viral genome replication	2	RSAD2/OAS3
BP	GO:0098586∼cellular response to virus	2	OAS3/CXCL10
BP	GO:0045069∼regulation of viral genome replication	2	RSAD2/OAS3
BP	GO:0048525∼negative regulation of viral process	2	RSAD2/OAS3
MF	GO:0016829∼lyase activity	2	RSAD2/CD38
MF	GO:0001664∼G protein-coupled receptor binding	2	RTP4/CXCL10
MF	GO:0008603∼cAMP-dependent protein kinase regulator activity	1	CXCL10
MF	GO:0050135∼NAD(P)+ nucleosidase activity	1	CD38
MF	GO:0061809∼NAD + nucleotidase, cyclic ADP-ribose generating	1	CD38
MF	GO:0045236∼CXCR chemokine receptor binding	1	CXCL10

Meanwhile, KEGG analysis showed that hub genes were associated with immune-related pathways, containing Hepatitis C, Influenza A, Epstein-Barr virus infection, and Coronavirus disease - COVID-19 (Figure 3E; Table 2). For the construction of a PPI network, 9 hub gens associated with macrophage M1 were updated in the STRING online tool. In-depth visualization was performed using Cytoscape software. The PPI network contained 9 genes, 9 nodes, and 21 edges (Figures 3B, C).

**TABLE 2:** KEGG pathway analysis of hub genes.

Pythway	Count	Fold enrichment	*p*-Value	Gene symbol
Hepatitis C	3	38.904	2.77E-05	RSAD2/OAS3/CXCL10
Influenza A	3	35.719	3.58E-05	RSAD2/OAS3/CXCL10
Epstein-Barr virus infection	2	20.158	0.00355	OAS3/CXCL10
Coronavirus disease - COVID-19	2	17.552	0.00467	OAS3/CXCL10

### Identification of DEGs

**FIGURE 4:**
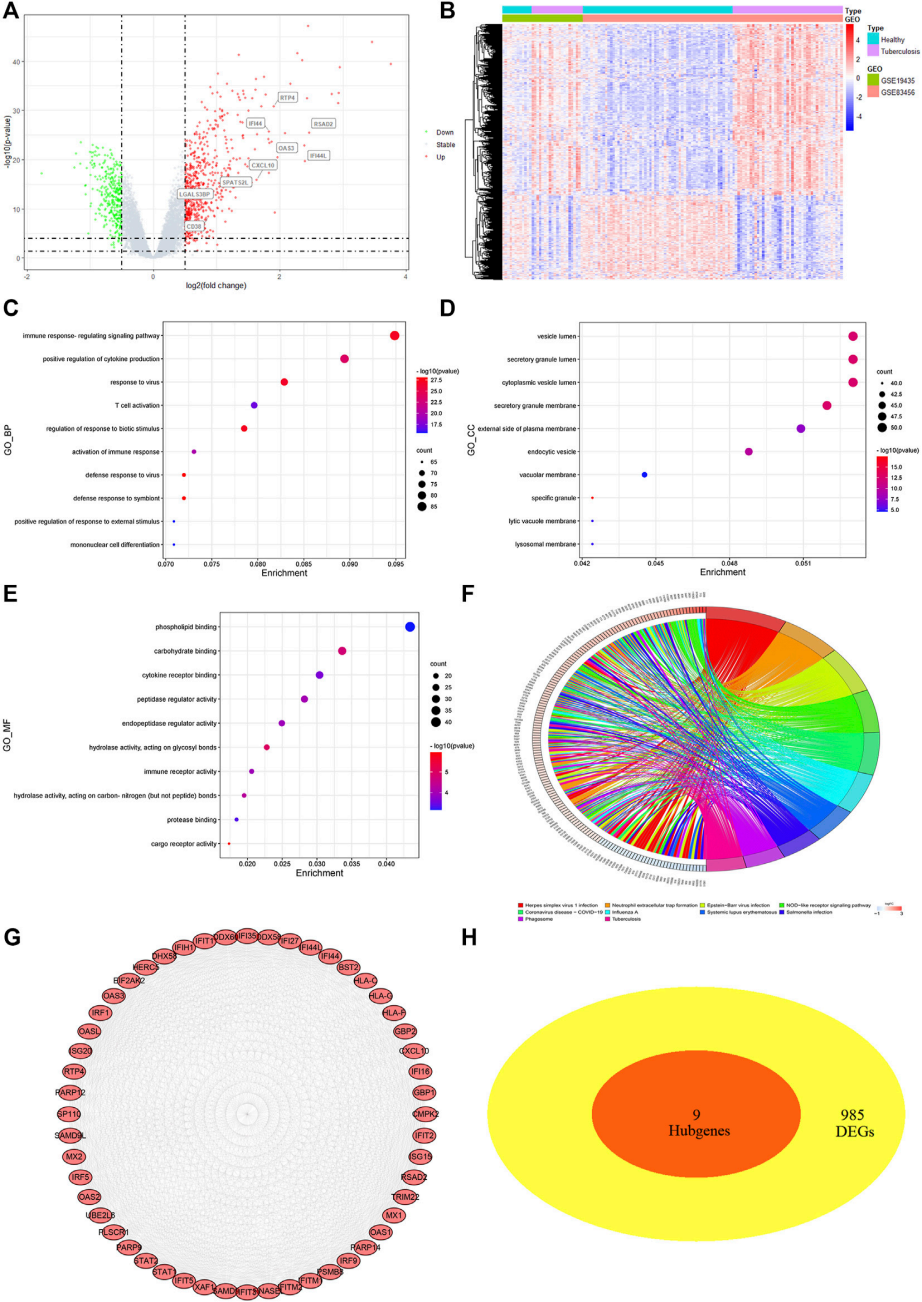
Differentially expressed genes (DEGs) screening and analysis in TB cases **(A)** Volcano plot presenting DEGs between TB patients and healthy individual. Red spot stood for 666 significant upregulated genes and green stood for 328 downregulated genes [FDR< 0.05 and |log FC|≥0.5]. **(B)** Heatmap of 994 DEGs filtered out *via* “limma” R package. In two dataset (GSE19435 and GSE83456), samples and genes were respectively sorted by columns and rows. Blue and purple squares comprised healthy and TB group, respectively. **(C–E)** Top 10 GO-BP, CC, MF items of DEGs. **(F)** Top 10 KEGG pathway of DEGs. **(G)** The top module extracted from PPI, red nodes stood for upregulated DEGs. **(H)** VENN diagram selected common genes between hub genes and DEGs that were described as candidate key genes.

Exact cutoff values (FDR <0.05 and |log FC|≥0.5) were used to investigate novel and reliable diagnostic biomarkers in TB patients. GSE19435 and GSE83456 yielded 994 DEGs (666 upregulated and 328 downregulated genes) (Supplementary Table S4). Two genes’ expression profiles’ heat maps and volcano plots revealed a consistent difference between normal and TB (Figures 4A, B). 994 DEGs were submitted for GO and KEGG analysis to investigate the biological mechanisms and cellular processes in TB patients. According to the top 10 GO terms, DEGs are primarily involved in immune signaling pathway regulation, cytokine production, T cell activation, secretory anti-inflammatory protein, immune receptor activity, and so on (Figures 4C–E). The KEGG term suggested that DEGs participate in the tuberculosis process *via* the NF- κB, TNF, T cell receptor, toll-like receptor, C-type lectin receptor signaling pathways, and so on (Figure 4F).

Furthermore, the STRING program was updated with DEGs, 958 of which were defined as significant DEGs in protein interaction. The Cytoscape software MCODE plug-in extracted the top modules of DEGs with 52 nodes and 1,097 edges based on degree value (Figure 4G). Due to the inability to identify key genes using different methods, we submitted the macrophage-related hub genes and DEGs to “VennDiagram” for consistent gene identification. SPATS2L, RTP4, RSAD2, OAS3, LGALS3BP, IFI44L, IFI44, CXCL10, and CD38 were all extracted (Figure 4H). Both were significantly upregulated in TB patients, with a *p*-value of <0.01 (Figure S1 D).

### Determination of key genes

To ensure the stability and reliability of the above results, an external dataset (GSE34608) was used to test the expression of candidate key genes, revealing that SPATS2L, RTP4, RSAD2, OAS3, IFI44L, IFI44, CXCL10, and CD38 were upregulated in TB patients compared to healthy controls.

**FIGURE 5:**
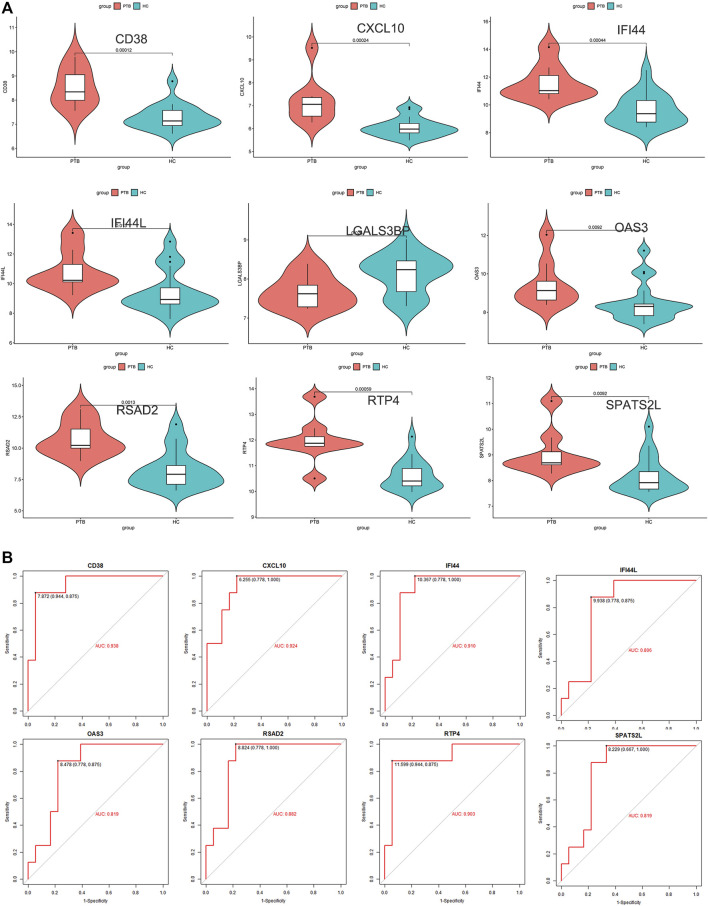
External validation. **(A)** Expression level of 9 candidate key genes between normal and TB samples were verified using external dataset (GSE34608). **(B)** ROC curve analyses for SPATS2L, RTP4, RSAD2, OAS3, IFI44L, IFI44, CXCL10, and CD38.

Only the level of LGALS3BP was reduced in the TB group (Figure 5A). ROC analysis has been commonly used to evaluate the accuracy of medical diagnostic tests (Zou et al., 2007). Except for LGALS3BP, candidate key genes were submitted for ROC analysis on an external database (GSE34608). Finally, the four genes with the highest performance in distinguishing between TB and normal samples were extracted, indicating that they can serve as key genes for further experimental verification. They were as follows: CD38 (AUC value = 0.938), CXCL10 (AUC value = 0.924), IFI44 (AUC value = 0.910), and RTP4 (AUC value = 0.903) (Figure 5B).

### Validation of key genes in *M. tuberculosis*-BCG infected macrophage

**FIGURE 6:**
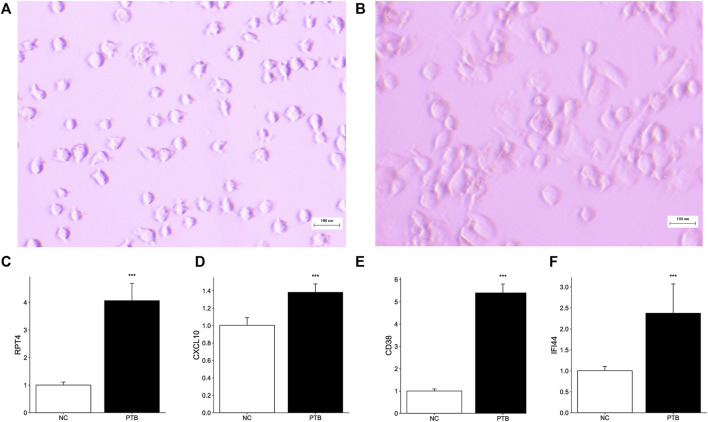
Effect of M. tuberculosis-BCG on THP-1 cells. The microscope images of **(A)** THP-1 cells, and **(B)** PMA successfully induced THP-1 cells adhered and differentiation. White scale bar, 100 μm. **(C–F)** THP-1 cells were infected with BCG for 24 h. Expression level of RTP4, CXCL10, CD38, and IFI44 were tested by qRT-PCR (Data are mean ± SD of 3 separate experiments, *p < 0.05, **p < 0.01, ***p < 0.001).

THP-1 is a human monocytic leukemia cell line that is commonly used to study macrophage response and mechanisms (Beckwith et al., 2020; Wu et al., 2022). PMA successfully induced THP-1 cell differentiation, resulting in mature macrophage-liking cells (Figures 6A, B). To validate the transcriptome information, we used qRT-PCR to investigate key gene activation in *M. tuberculosis*-BCG infected differentiated THP-1 cells. Notably, all key genes are upregulated in the TB group, implying that they have the potential to be effective diagnostic biomarkers for TB (Figures 6C–F).

### Novel small molecule therapeutic agents targeting the biological function in TB

**FIGURE 7:**
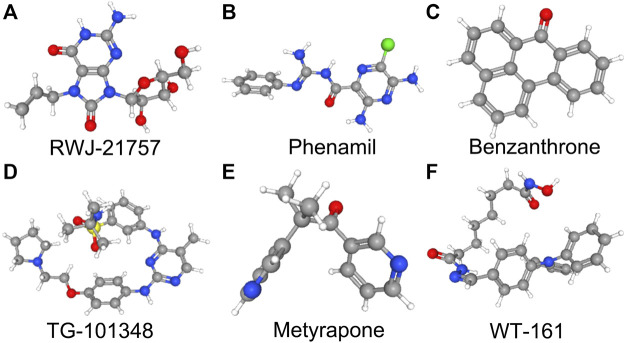
3D structures of small molecular compounds. To reveal the promising compounds, 300 DEGs were submitted to Connectivity Map (CMap) (https://clue.io/), and the cutoff criterion (0.8<|connectivity score|<1 and *p* < 0.05) was used. Top 6 small molecular compounds with highest enrichment score were identified as promising drugs that may have potential therapeutic effects against TB.

We submitted DEGs to the CMap database to find the underlying small-molecule therapeutic agents for tuberculosis. Based on significant enrichment value, 10 small molecules capable of suppressing DEGs expression of TB were identified, 6 of which were considered potential therapeutic compounds (RWJ-21757, WT-161, phenamil, metyrapone, benzanthrone, and TG-101348) (Table 3). Both may be involved in regulating target gene expression and have a therapeutic impact on TB. Figure 7 depicts the 3D structure of a small molecule.

**TABLE 3:** The result of CMap.

Name	Score	Description	Target
RWJ-21757	91.71	TLR agonist	TLR7
phenamil	89.36	TRPV antagonist	PKD2L1
benzanthrone	86.87	Aromatic hydrocarbon derivative	
TG-101348	−88.24	FLT3 inhibitor	JAK2, FLT3, BRD4, JAK1, JAK3, RET, TYK2
metyrapone	−88.81	Cytochrome P450 inhibitor	CYP11B1, HSD11B1, NR3C2
WT-161	−92.36	HDAC inhibitor	HDAC6

## Discussion

TB is a contagious chronic disease caused by Mtb that primarily affects the lungs, resulting in severe hemoptysis and fever (Fogel 2015; Orazulike et al., 2021). Recent research has shown that macrophage dysregulation is crucial in determining the occurrence, development, and prognosis of tuberculosis (TB) (Pal et al., 2021). Investigation of immune response-related genes remains a potent tool for identifying the TB susceptibility mechanism (Gopalaswamy et al., 2020). In this pilot study, we extracted gene expression data from two mRNA profiles obtained from TB and normal blood samples. The M1 macrophage infiltration model revealed 9 hub genes. Further investigation revealed 4 of the 9 hub genes were identified as reliable candidate biomarkers with significantly higher detection levels in TB samples. Meanwhile, 6 small molecules were predicted to be potential drugs targeting tuberculosis’s biological function. WGCNA was used to establish diverse models by selecting 2,413 variant genes and macrophage infiltration levels in TB samples. Correlation coefficients were used to identify the most important macrophage M1-related modules. With a cutoff value of (MM > 0.8 and GS > 0.5), 9 of 120 genes in hub models were chosen as hub genes.

The enrichment analysis results suggested that hub genes are strongly linked to the tuberculosis immune response. SPATS2L, RTP4, RSAD2, OAS3, LGALS3BP, IFI44L, IFI44, CXCL10, and CD38 were identified as consistent genes between DEGs and hub genes that act as candidate key genes. In TB samples from GSE19435 and GSE83456, 9 candidate key genes showed significantly increased expression. An external mRNA profile (GSE34608) was used to perform expression analysis, which revealed that only LGALS3BP was downregulated, while other candidate key genes were significantly upregulated in TB patients.

ROC analysis was performed, and the AUC area was evaluated in turn. The top four genes with the highest AUC value were identified as hub genes for additional experimental validation. Finally, qRT-PCR was used to examine the relative transcription levels of key genes in normal and TB-infected THP-1 cells, and the mRNA expression trends of key genes were consistent with bioinformatics data. These findings suggest that the 4 key genes are linked to the progression and diagnosis of tuberculosis.

Recent documents have revealed 4 key genes as guardians who actively participate in the protective immunity of various inflammatory diseases and cancer (Li et al., 2021a). IFI44 is found on human chromosome 1p31.1 ad belongs to the interferon-stimulated gene (ISG), which plays a significant role in immunoregulation and tumor cell recognition (Lukhele et al., 2019; Wang et al., 2020; Li et al., 2021b).

Its homologous gene, IFI44L, has been shown to promote macrophage differentiation and inflammatory cytokine secretion during Mtb infection (Jiang et al., 2021). In contrast, the precise role of IFI44 in tuberculosis has yet to be revealed. CXCL10 (C-X-C motif chemokine ligand 10) belongs to the CXC chemokine family. It can bind to CXCR3, triggering innate immune cell migration and regulating adhesion molecule expression, implying a significant role in immune cell development (Smit et al., 2003; Gao et al., 2009). In the meantime, CXCL10 overexpression has been repeatedly observed in tuberculosis patients (Bhattacharyya et al., 2018).

CD38 is a protein-coding gene that encodes a multifunctional glycoprotein found on the surface of immune cells (Mehta et al., 1996). It is also an effective diagnostic marker in various immune-related diseases such as tuberculosis and leukemias (Malavasi et al., 2008; Acharya et al., 2021). RTP4 (Receptor Transporter Protein 4) is a member of the RTPs family that is directly involved in modulating the expression of cell-surface G-coupled protein receptors (Saito et al., 2004; Boys et al., 2020). Several studies have found that RTP4 is strongly linked to a virus defense response and cancer prognosis (Li et al., 2021a). Following Lipinski’s rule-of-five for drug likeliness, a protein’s druggability is solely determined by its affinity and specificity for small molecules (Abi et al., 2017). Several bioinformatic tools that can prescreen candidate drugs in less time than traditional approaches have emerged (Xia 2017).

Using CMap, we discovered latent therapeutic small molecular compounds in tuberculosis. CMap yielded 6 compounds with higher enrichment scores: RWJ-21757, WT-161, phenamil, metyrapone, TG-101348, and benzanthrone. RWJ-21757 is a toll-like receptor (TLR)7 selective agonist with diverse immunobiological activities (Yu et al., 2022). It significantly improves innate immune responses by activating specific immune cells such as macrophages, T cells, and B cells (Goodman 1995). WT-161 is a potent histone deacetylase 6 (HDAC6) inhibitor widely used in cancer treatment by targeting the expression of CD38 (Garcia-Guerrero et al., 2021; Yu et al., 2022). Phenamil is an amiloride derivative involved in cell differentiation and primarily acts as a sodium channel blocker in various diseases (Garvin et al., 1985; Price et al., 2017). Metyrapone, a bipyridyl compound, is a reversible inhibitor of cytochrome P450. It contributes to inflammatory responses by suppressing endogenous adrenal corticosteroid synthesis and lowering glucocorticoid levels (Fantuzzi et al., 1993). TG-101348 is an ATP-competitive Janus kinase 2 (JAK2) inhibitor with antitumor activity by inducing cancer cell apoptosis (Wernig et al., 2008; Verstovsek 2009). Benzanthrone is an aromatic hydrocarbon derivative immunotoxic and can cause an inflammatory response. It is widely used in antimicrobial research (Tewari et al., 2015; Tsanova et al., 2020).

## Conclusion

To explore M1 macrophage-related tuberculosis biomarkers, we first try using WGCNA and CIBERSORT algorithms in the current study. RTP4, CXCL10, CD38, and IFI44 were the 4 key genes that were confirmed by validating integrated information and experiments. Those of them were upregulated genes, that could act effective biomarkers and key therapeutic target genes. Additionally, RWJ-21757, WT-161, phenamil, metyrapone, TG-101348, and benzanthrone were potential small-molecule drugs for treating tuberculosis. Our research provides a novel viewpoint on immune and molecular TB diagnosis. Our research data has limitations. More clinical sample data and additional research are required to confirm the underlying mechanism of key genes and targeted drugs in tuberculosis.

## Data Availability

The datasets presented in this study can be found in online repositories. The names of the repositories and accession number can be found in the article/Supplementary Material.

## Supplementary material

SUPPLEMENTARY FIGURE S1 Training dataset (GSE19435 and GSE83456) analysis.**(A)** The boxplot of original data. **(B)** The boxplot showed a batch-normalized microarray profile using “sva” and “limma” packages. **(C)** The dendrogram and heatmap indicated the degree of infiltration of 3 types of macrophages in samples. Red stood for high degree of infiltration. **(D)** Differential expression of 9 candidate key genes between TB and normal samples.
