# Supplementary figures and images for: Integrated bioinformatic analyses investigate macrophage-M1-related biomarkers and tuberculosis therapeutic drugs
Source: Front Genet. 2023 Feb 8;14:1041892. doi: 10.3389/fgene.2023.1041892 (PMC9945105; doi:10.3389/fgene.2023.1041892)

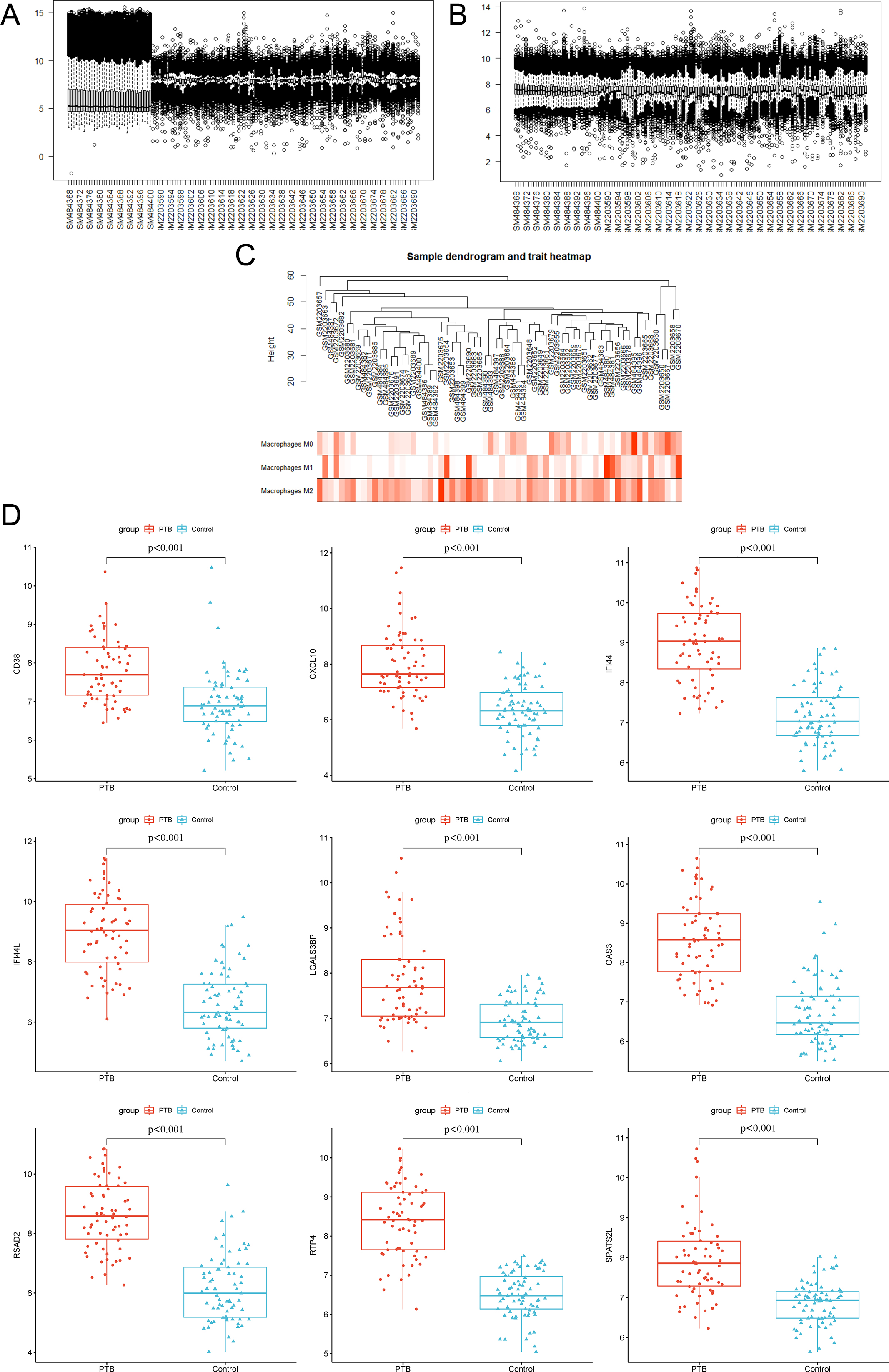

Supplement: Supplementary file 5 [file Image1.jpg]
